# Connectivity Effect on Electronic Properties of Azulene–Tetraazapyrene Triads

**DOI:** 10.3390/molecules31010002

**Published:** 2025-12-19

**Authors:** Xinyi Liu, Souren Majani, Jian Zhang, Simon M. Langenegger, Silvio Decurtins, Ulrich Aschauer, Shi-Xia Liu

**Affiliations:** 1 Department of Chemistry, Biochemistry and Pharmaceutical Sciences, W. Inäbnit Laboratory for Molecular Quantum Materials, WSS-Research Center for Molecular Quantum Systems, University of Bern, Freiestrasse 3, 3012 Bern, Switzerland; xinyi.liu@students.unibe.ch (X.L.); jian.zhang@unibe.ch (J.Z.); simon.langenegger@unibe.ch (S.M.L.); silvio.decurtins@unibe.ch (S.D.); 2 Department of Chemistry and Physics of Materials, University of Salzburg, Jakob-Haringer-Str. 2A, 5020 Salzburg, Austria; souren.majani@plus.ac.at

**Keywords:** azulene, cyclic voltammetry, intramolecular charge transfer, TD-DFT calculations, UV-Vis spectroscopy, optical pH sensors, tetraazapyrene

## Abstract

Azulene-based chromophores are of growing interest due to their unique electronic structures and potential applications as pH-responsive optical materials. In this study, a series of azulene–1,3,6,8-tetraazapyrene (TAP) triads were successfully synthesized and characterized to systematically explore how connectivity between the TAP and azulene units influences their optical and redox properties. UV-Vis absorption spectroscopy and cyclic voltammetry measurements clearly show that the electronic properties depend heavily on the connectivity pattern, as the effective π-conjugation and molecular planarity vary considerably in triads. Remarkably, triads **A_22_** and **A_26_**, in which the TAP core is directly connected through the electron-rich five-membered ring, exhibit enhanced π-conjugation and pronounced color changes upon protonation. In contrast, **A_66_**, linked via the electron-deficient seven-membered ring, reveals weaker π-conjugation and less pronounced pH-responsiveness. These experimental findings are further supported by DFT calculations. This comprehensive structure–property relationship study provides valuable insights for the rational design of advanced optoelectronic and stimuli-responsive materials.

## 1. Introduction

Organic functional materials consisting of π-electron-donating (D) and -accepting (A) groups have attracted considerable interest due to their unique optoelectronic properties, which render them highly promising in a variety of applications such as organic electronics [1,2,3,4], molecular switches and sensors, bioimaging [5], and photo-therapy [6]. The distinctive electronic behavior of these materials arises from the interaction between the D and A subunits, which facilitates intramolecular charge transfer (ICT) in the visible spectral range. Therefore, careful molecular design, including chemical modification of the D and A moieties, as well as the nature of the linker connecting them, can achieve precise and efficient control of the optoelectronic properties. Herein, we describe the synthetic pathway of a series of azulene–tetraazapyrene (TAP) triads in which the azulene units are directly connected with the central TAP core through the carbon atoms in position 2 or 6. Depending on the substitution pattern, the dominant electronic transition can shift between a localized π–π* transition and an ICT transition.

Azulene is an isomer of naphthalene distinguished by its non-hexagonal π-structure, composed of fused five- and seven-membered rings. This unique geometry gives rise to a deep blue color, anti-Kasha fluorescence, and a large dipole moment [7,8]. Its intrinsic electronic polarization endows azulene with pH-responsive properties, allowing it to act as a pH sensor that exhibits distinct changes in optical absorption upon protonation or deprotonation [9,10,11,12]. It is frequently incorporated into π-conjugated frameworks, contributing to high-performance semiconducting materials. The stimuli-responsive behavior and the optoelectronic performance of these π-functional materials can be effectively modified by structural extension at the 2- and/or 6-position on the azulene units [13,14,15,16,17,18,19].

On the other hand, a nitrogen-rich analogue of pyrene, 1,3,6,8-tetraazapyrene (TAP), features a planar and highly electron-deficient π-conjugated core owing to the incorporation of four nitrogen atoms into the aromatic system. This modification lowers the LUMO energy level and narrows the HOMO–LUMO gap, leading to a red-shift of optical transitions and enhanced redox activity. Consequently, TAP is recognized as an excellent n-electron acceptor in D–A conjugates for potential applications in organic semiconductors, optoelectronic devices, and supramolecular systems [20,21,22,23,24,25,26,27]. However, TAP-functionalized materials remain largely unexplored due to synthetic challenges. Recent advances in regioselective cascade halogenation and functionalization strategies have enabled the synthesis of structurally diverse TAP derivatives with enhanced solubility and tunable electronic interactions [28,29]. In particular, substitution at the 2,7-positions with bulky groups such as *tert*-butyl has been shown to improve solubility while preserving the intrinsic electronic characteristics of TAP.

**Scheme 1:**
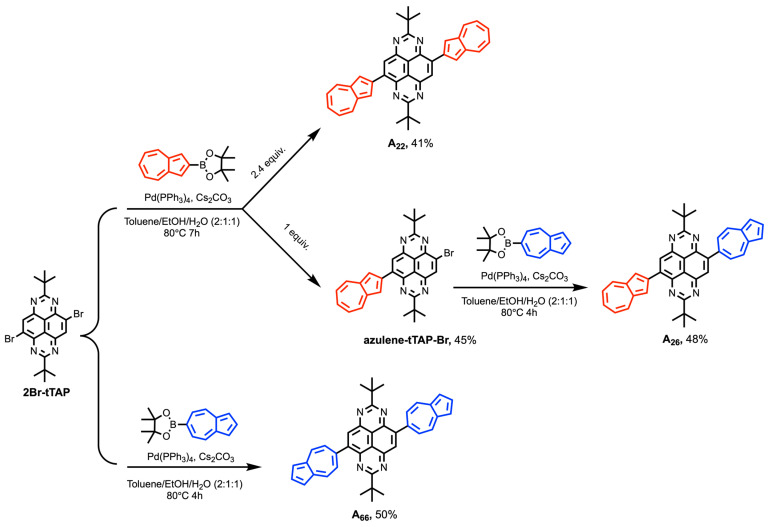
Synthetic routes towards azulene–TAP triads **A_22_**, **A_26_**, and **A_66_**.

In this work, we designed and synthesized a series of azulene–TAP conjugates featuring either symmetric or asymmetric substitution patterns on the azulene moieties. As illustrated in Scheme 1, triads **A_22_** and **A_66_** represent symmetric structures, where two azulene rings are directly linked to the TAP core at the 2- and 2-positions and the 6- and 6-positions, respectively. In contrast, triad **A_26_** exhibits an asymmetric configuration, with one azulene ring attached to the TAP core at the 2-position and the other at the 6-position. These newly prepared triads were fully characterized by NMR, HRMS, UV-Vis spectroscopy, and electrochemical analysis. Their acid–base-responsive properties with instant color switching behavior were probed through titration with trifluoroacetic acid and triethylamine, while time-dependent density functional theory (TD-DFT) calculations provided insights into their electronic transitions. By comparing the photophysical and electrochemical behavior of these three compounds, we reveal the influence of connectivity on the degree of ICT, HOMO–LUMO energy band gap, and pH sensitivity.

## 2. Results and Discussion

### 2.1. Synthesis

As depicted in Scheme 1, a series of azulene-incorporated TAP derivatives (**A_22_**, **A_26_**, **A_66_**) were synthesized in moderate yields (41–50%) via Suzuki–Miyaura cross-coupling reactions of **2Br-tTAP** [28] with 2-(4,4,5,5-tetramethyl-1,3,2-dioxaborolan-2-yl)azulene [30] and/or 6-(4,4,5,5-tetramethyl-1,3,2-dioxaborolan-2-yl) azulene [31]. The reactions were catalyzed by Pd(PPh_3_)_4_ and Cs_2_CO_3_ in a mixed solvent system (toluene/H_2_O/EtOH, 2:1:1) under argon at reflux. The symmetric triads **A_22_** and **A_66_** were obtained by reacting **2Br-tTAP** with a slight excess (2.4 equivalents) of the corresponding azulene boronic ester. In contrast, the asymmetric **A_26_** was synthesized via a two-step route involving the formation of an intermediate, **azulene-tTAP-Br**, synthesized by the reaction of **2Br-tTAP** with 1 equivalent of 2-substituted azulene boronic ester. This intermediate was subsequently reacted with 1 equivalent of 6-substituted azulene boronic ester under similar conditions to afford **A_26_**. All products were purified by column chromatography on silica gel and fully characterized by ^1^H NMR spectroscopy and High-resolution Mass Spectroscopy to confirm their structures.

### 2.2. Optical Properties

**Figure 1:**
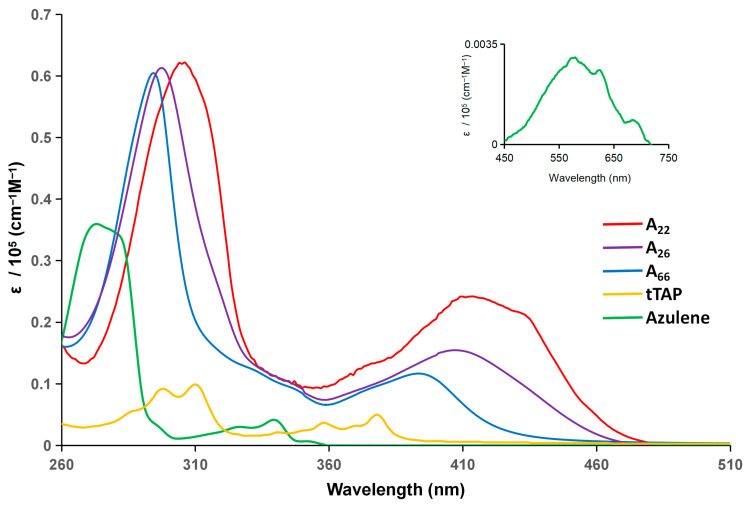
UV-Vis absorption spectra of **A_22_** (red), **A_26_** (purple), **A_66_** (blue), **tTAP** (yellow), and azulene (green); (c = 1 × 10^−5^ M) in CH_2_Cl_2_ at r.t.

The optical properties of the three triads in CH_2_Cl_2_ were systematically investigated using UV-Vis absorption spectroscopy. As shown in Figure 1 and Table S1, all three compounds strongly absorb in the UV-visible spectral region. Compared to the reference compounds azulene (green curve) and 2,7-di-*tert*-butyl-tetraazapyrene (**tTAP**) (yellow curve), the intense absorption band observed in the UV region for each triad is characteristic of π–π* transitions, associated with azulene–TAP moieties. Moreover, a broad and strong absorption band appears around 390 nm for **A_66_**, 400 nm for **A_26_**, and 410 nm for **A_22_**, clearly distinct from the multiple absorption peaks of the individual **tTAP** component. Consequently, it reflects extended π-conjugation between the azulene and TAP units and partial CT-type transitions characteristic of the triads. Notably, this lowest-energy absorption band becomes more pronounced and is progressively red-shifted going from **A_66_** to **A_26_** to **A_22_**, indicating that the extent of π-conjugation between the TAP core and azulene units is significantly influenced by the connectivity pattern. When the azulene units are linked to the central TAP core through the carbon atom at the 2-position, electronic communication is more efficient due to enhanced π-delocalization, which arises from improved coplanarity between the TAP and azulene cores.

**Figure 2:**
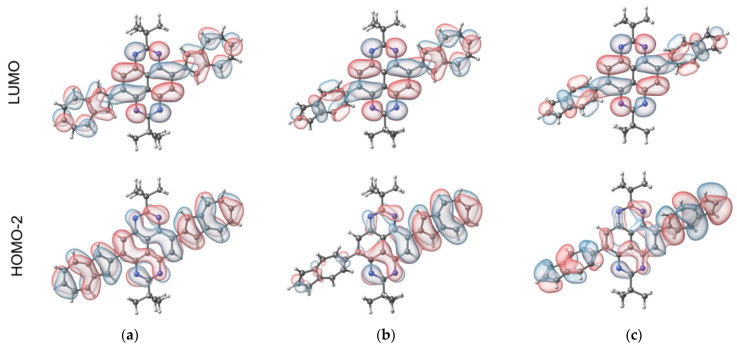
DFT-calculated frontier molecular orbitals of **A_22_** (**a**), **A_26_** (**b**), and **A_66_** (**c**) that are involved in the electronic transitions in the visible spectral region.

To rationalize the electronic absorption spectra of the triads, a detailed computational study was performed, with detailed results shown in Tables S3–S5 and Figures S4 and S5. As depicted in Figure S5, the HOMO and HOMO-1 are exclusively localized on the azulene skeleton for all compounds. In the case of **A_22_**, the HOMO-2 and HOMO-3 become more delocalized with significant amplitude over both azulene moieties. For **A_26_**, the HOMO-2 is delocalized across the TAP and 2-substituted azulene ring while the HOMO-3 is primarily localized on the 6-substituted azulene ring. In stark contrast, the HOMO-2 and HOMO-3 are nearly degenerate and confined to the azulene ring in **A_66_**. Interestingly, the LUMO is mainly delocalized over the TAP core and the azulene rings in **A_22_**. In **A_26_**, it is localized on the TAP core and extends to the 2-substituted azulene ring, whereas in **A_66_**, it is exclusively localized on the TAP unit. This spatial distribution of molecular orbitals is in good agreement with the D–A characteristics of the triads. The change in connectivity from the 2- to the 6-position results in reductions in both HOMO and LUMO energies. However, the HOMO-2 (see Figure 2 for transition-dominating molecular orbitals), which is predominantly involved in the lowest-energy electronic transition (448.6 nm, H-2 → LUMO (72%), f = 0.3700 for **A_22_**; 455.4 nm, H-2 → LUMO (81%), f = 0.2699 for **A_26_**; λ = 436.5 nm, H-2 → LUMO (82%), f = 0.2879 for **A_66_**), is more significantly affected, giving rise to a hypsochromic shift in the absorption spectra going from **A_22_** to **A_26_** to **A_66_**, which aligns well with the experimental results (Figure 1).

### 2.3. pH-Responsiveness

Initial pH-responsiveness studies revealed that compounds **A_22_**, **A_26_**, and **A_66_** undergo reversible protonation–deprotonation accompanied by distinct changes in their absorption spectra upon exposure to trifluoroacetic acid (TFA) and triethylamine (TEA), respectively. These observations demonstrate that azulene–TAP triads act as effective pH-sensitive optical probes.

**Figure 3:**
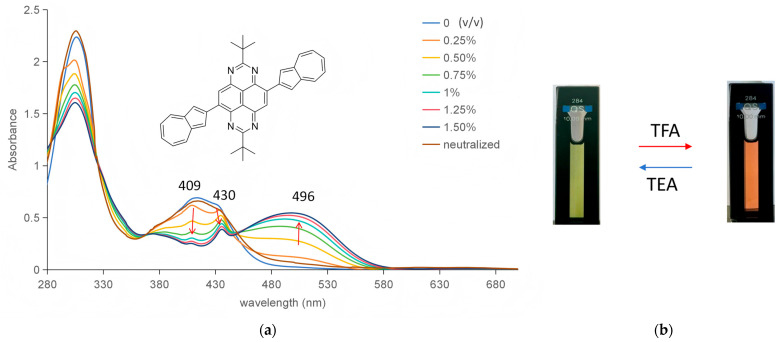
The spectral variation (**a**) and solution color evolution (**b**) of **A_22_** at a concentration of 10^−5^ M upon successive addition of TFA.

**Scheme 2:**
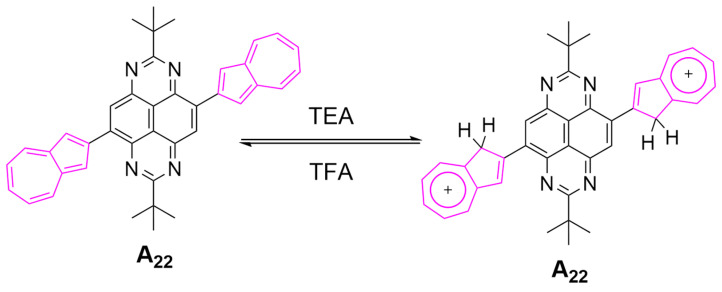
The formation of an azulenium dication of triad **A_22_** upon protonation and reversible proton-responsiveness by neutralization in the presence of TEA.

To examine the influence of molecular structure on the acid–base-responsive optical properties, the UV-Vis absorption spectra of **A_22_** in CH_2_Cl_2_ were recorded during a stepwise titration with TFA. As shown in Figure 3, the absorption band at 304 nm continuously decreased in intensity upon addition of TFA. Concurrently, the bands at 409 nm and 430 nm diminished, while a distinct and broad absorption band emerged at 496 nm, accompanied by an isosbestic point at 445 nm. This observation indicates a well-defined two-state equilibrium between the neutral and protonated species, consistent with the formation of the protonated azulenium species, as illustrated in Scheme 2. Remarkably, the lowest-energy absorption band extended up to 600 nm, which can be attributed to a back CT from the HOMO-1 localized on the TAP core to the LUMO on the azulenium units (Figure S5). These spectral changes were accompanied by a visible color transition from green to orange. When the TFA concentration reached 1.5% (*v*/*v*), no further spectral evolution was detected, implying complete protonation. Subsequent addition of an equivalent amount of TEA nearly restored the original spectral profile (wine red curve, Figure 3).

Upon gradual addition of TFA, both **A_26_** and **A_66_** also exhibited notable spectral changes (Figures S1 and S2) indicative of acid-induced protonation and modulation of the electronic structure. In contrast to that for **A_22_** (Figure 1), the initial absorption peak at 403 nm in **A_26_** rapidly decreased with the concomitant appearance of a wide absorption band from 400 to 700 nm (Figure S1), as evidenced by a color change of the solution from greenish yellow to pink. The absorption band at 393 nm in **A_66_** gradually decreased, accompanied by the appearance of a new, weak, and broad peak at 443 nm (Figure S2), leading to a slight color change upon protonation. Based on the onset of the lowest-energy absorption band, the optical HOMO–LUMO band gaps were estimated to be 2.11 eV for **A_22_**-2H^+^, 2.06 eV for **A_26_**-2H^+^, and 2.24 eV for **A_66_**-2H^+^, which are lower than those for the neutral states (2.59 eV for **A_22_**, 2.63 eV for **A_26_**, and 2.88 eV for **A_66_**). As a result, the observed bathochromic shift of the lowest-energy absorption band in these triads is attributed to a decrease in the HOMO–LUMO gap upon protonation. These distinct differences in spectral evolution reflect how substitution of azulene at the 2- versus the 6-position affects acid–base-responsive behaviors.

Similarly to that of **A_22_**, neutralization of acidic solutions of **A_26_** and **A_66_** with TEA regenerated the original absorption spectra corresponding to their neutral state (Figures S1 and S2). This reversible behavior renders them promising in the development of novel molecular probes enabling the detection of even weak acid and base with the naked eye.

**Figure 4:**
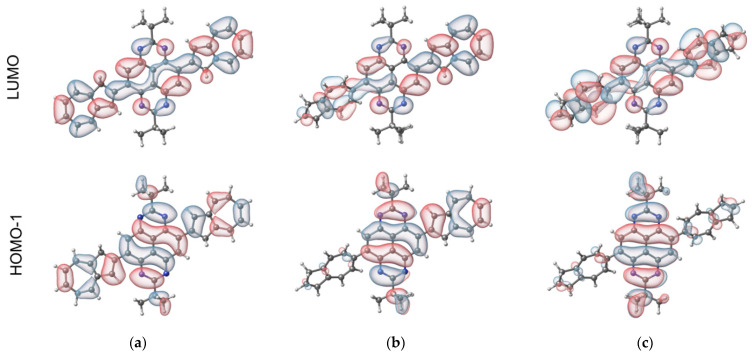
DFT-calculated frontier molecular orbitals of protonated **A_22_·2H^+^** (**a**), **A_26_·2H^+^** (**b**), and **A_66_·2H^+^** (**c**) that are involved in the electronic transitions in the visible spectral region.

To gain deeper insight into the changes in the UV-visible absorption spectra, DFT calculations were also carried out on the protonated species. Upon protonation of azulene rings to form the corresponding azulenium cation, both the HOMO and LUMO energy levels are significantly lowered (Figure S5 versus Figure S7). Notably, the HOMO-1 displays a large amplitude on the central TAP core with an increasing contribution from the 2-substituted azulenium ring(s) as the connectivity shifts from the 6- to the 2-position. This shift leads to decreased stabilization of the HOMO-1 in the order **A_66_** ⟶ **A_26_** ⟶ **A_22_**. The LUMO, on the other hand, is now predominantly located on the azulenium rings in all three cases and largely unaffected by connectivity. The lowest-energy electronic transition is primally assigned to a back CT from the HOMO-1 localized on the TAP core to the LUMO on the azulenium units (see Figure 4 for these transition-dominating orbitals). As a result, this transition undergoes a progressive bathochromic shift following the order **A_66_** ⟶ **A_26_** ⟶ **A_22_**, which matches well with experimental observations (Figure 3, Figures S1 and S2).

### 2.4. Electrochemical Properties

**Figure 5:**
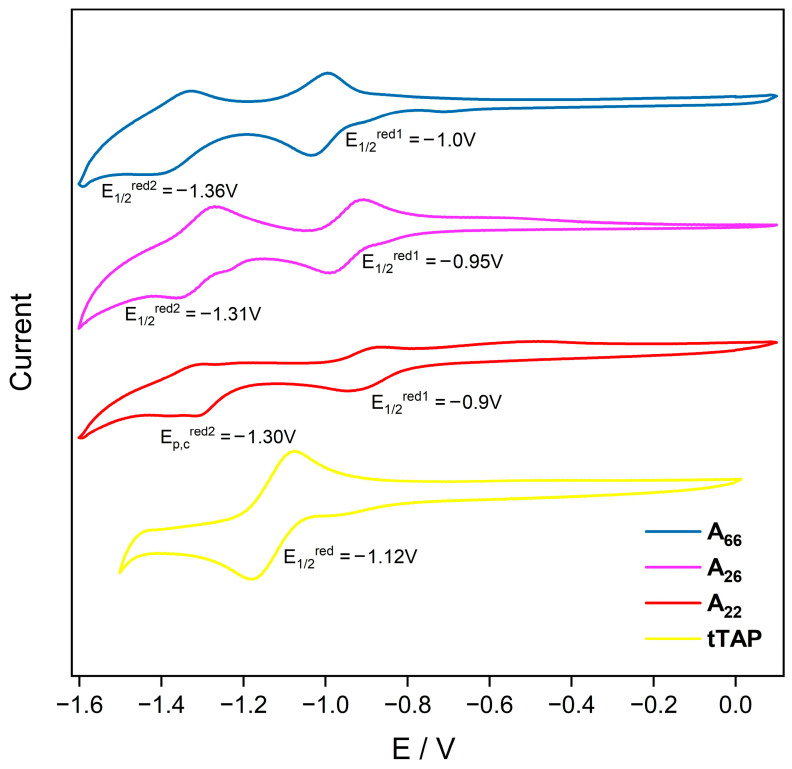
Cyclic voltammograms of **A_22_**, **A_26_**, **A_66_**, and **tTAP** were measured in dichloromethane solution (10^−5^ M) containing 0.1 M TBAPF_6_ as the supporting electrolyte at r.t., with a Pt working electrode and Ag/AgCl reference electrode at a scan rate of 100 mV s^−1^.

Cyclic voltammetry (CV) measurements in dichloromethane were performed to investigate the electronic properties of the triads. The redox potentials of the triads, along with those of reference compound **tTAP** and azulene, as well as the estimated HOMO and LUMO energy levels, are summarized in Table S2. As shown in Figure 5, all triads except **A_22_** undergo two reversible reduction processes (*E*_1/2_^red1^, *E*_1/2_^red2^), which correspond to successive single-electron reductions of the TAP core. In contrast, the second reduction of **A_22_** becomes less reversible, most likely due to partial aggregation. By comparison, **tTAP** shows only one reversible reduction wave (*E*_1/2_^red^ = −1.12 V vs. Ag/AgCl). The first reduction potentials of triads **A_22_** and **A_26_** are shifted anodically by ca. 0.22 V and 0.17 V, respectively, relative to that of **tTAP**, which likely arises from the effective extension of π-conjugation of the TAP core through the electron-rich five-membered ring of the 2-substituted azulene unit, facilitated by their coplanar geometry as shown in the LUMO (Figure 2). Similarly, **A_66_** exhibits an anodic shift of the first reduction potential by 0.12 eV, which is attributed to the electron-withdrawing effect of the electron-deficient seven-membered ring in 6-substituted azulene moieties. In the anodic region (Figure S3), each triad displays a broad featureless oxidation peak (*E*_pa_) corresponding to the irreversible oxidation of azulene units. Compared to azulene (+1.18 V vs. Ag/AgCl), **A_22_** and **A_26_** oxidize at slightly lower potentials, whereas **A_66_** oxidizes at nearly the same potential. The easier oxidation for **A_22_** and **A_26_** again reflects the influence of extended π-conjugation, while in **A_66_** the large torsion angle between the TAP and azulene units suppresses electronic communication. All these results clearly demonstrate that the connectivity between the central TAP and azulene rings strongly influences the redox behavior of the triads. The differences arise from variations in effective π-conjugation, which depend on whether the TAP core is directly linked to the electron-rich five-membered or the electron-deficient seven-membered ring of the azulene, leading to planar or nonplanar configurations, respectively.

On the basis of the onsets of the first reduction and oxidation waves, the LUMO and HOMO were estimated (Table S2). The HOMO energy levels of **A_22_** (–5.16 eV), **A_26_** (–5.14 eV), and **A_66_** (–5.15 eV) are slightly higher than that of azulene itself (−5.32 eV), while their LUMO energy levels (**A_22_**: –3.47 eV; **A_26_**: –3.44 eV; **A_66_**: –3.48 eV) are lower than that of **tTAP** (–3.22 eV). It has been demonstrated that the incorporation of azulene rings into the TAP core results in LUMO stabilization and HOMO destabilization, which is in good agreement with DFT calculations (Figure 2).

## 3. Materials and Methods

All commercial chemicals and reagents, unless otherwise stated, were used without further purification. All the synthetic experiments were performed under a dry argon atmosphere using standard Schlenk techniques unless otherwise mentioned. 4,9-Dibromo-2,7-di-*tert*-butyl-tetraazapyrene (**2Br-tTAP**) [28], 2-(4,4,5,5-tetramethyl-1,3,2-dioxaborolan-2-yl) azulene [30], and 6-(4,4,5,5-tetramethyl-1,3,2-dioxaborolan-2-yl)azulene [31] were prepared as described in the literature.

^1^H NMR and ^13^C NMR spectra were recorded on Bruker Avance 300 (300 MHz) and 400 (400 MHz and 101MHz) spectrometers (Bruker, Fällanden, Switzerland). Chemical shifts (*δ*) are reported in parts per million (ppm) and are referenced to the residual solvent peak (CD_2_Cl_2_: (δ ^1^H = 5.32 ppm, δ ^13^C = 53.84 ppm); CDCl_3_: (δ ^1^H = 7.26 ppm, δ ^13^C = 77.16 ppm)). The coupling constants (*J*) are listed in Hertz (Hz). The terms s., d., t., and m. indicate singlet, doublet, triplet, and multiplet, respectively; dd is a doublet of doublets; dt is a doublet of triplets. Mass spectra were measured by the Analytical Research and Services (ARS) of the University of Bern, Switzerland, on a Thermo Fisher LTQ Orbitrap XL (Waltham, MA, USA) using Nano Electrospray Ionization (nano-ESI). UV-Vis spectra were measured on a Jasco V-730 spectrophotometer (Tokyo, Japan) using quartz cuvettes with an optical path of 1 cm.

Cyclic voltammetry (CV) measurements were performed using a typical one-compartment, three-electrode setup driven by a Metrohm PGSTAT101 potentiostat (Herisau, Switzerland). A three-electrode cell equipped with a disk Pt working electrode (diameter of 3 mm), a glassy carbon counter-electrode, and a Ag/AgCl reference electrode filled with a solution of 2 M LiCl in ethanol was used. The electrochemical experiments were carried out under an oxygen-free atmosphere in dichloromethane with TBAPF_6_ (0.1 M) as a supporting electrolyte. Under these conditions, ferrocene exhibits a reversible oxidation peak with an *E*_1/2_ of 0.53 V.

DFT calculations were performed using NWChem 7.0.2 [32], at the B3LYP/6-31G** level of theory. Geometries were relaxed to within default criteria, starting from 5 configurations per molecule and retaining the lowest-energy one. TD-DFT calculations of the absorption spectra were carried out for each lowest-energy configuration, solving for 40 excited states. The effect of pH was modeled by protonation, which was carried out by adding a hydrogen to each of the two five-membered rings and adjusting the total charge to +2. All calculations shown in the manuscript were carried out in gas phase, without the inclusion of an implicit solvation model. Such a model will lead to shifts in absolute peak positions while retaining relative shifts as shown in Figure S6.

Synthesis of **A_22_**. In a 50 mL two-necked flask, 50 mg of **2Br-tTAP** (0.105 mmol), 64 mg of 2-(4,4,5,5-teramethyl-1,3,2-dioxaborolan-2-yl)azulene (0.25 mmol), 205 mg of Cs_2_CO_3_ (0.63 mmol), and 7 mg of Pd(PPh_3_)_4_ (0.006 mmol) were suspended in a mixed solvent of toluene/H_2_O/EtOH (10 mL/5 mL/5 mL) under argon. The mixture was heated at 80 °C under Ar for 7 h. After cooling down to room temperature, the reaction was quenched with 0.6 mL of a 2M HCl aqueous solution, extracted with toluene, and washed with water and brine. The organic layer was dried over Na_2_SO_4_, filtered, and concentrated under reduced pressure. The obtained crude product was purified by column chromatography on silica gel (CH_2_Cl_2_/hexane = 1:2 to 1:1, *v*/*v*) to afford **A_22_** as a green powder (25 mg, 41%). ^1^H NMR (400 MHz, CD_2_Cl_2_) *δ* 9.13 (s, 2H), 8.60 (s, 4H), 8.55–8.51 (m, 4H), 7.66 (dd, *J* = 10.2 Hz, 2H), 7.27 (dd, *J* = 9.9 Hz, 4H), 1.78 (s, 18H). HR–MS (ESI, positive): *m*/*z* calcd for [M + H]^+^ 571.2856, found 571.2853.

Synthesis of **A_66_**. In a 50 mL two-necked flask, 50 mg of **2Br-tTAP** (0.105 mmol), 64 mg of 6-(4,4,5,5-teramethyl-1,3,2-dioxaborolan-2-yl)azulene (0.25 mmol), 205 mg of Cs_2_CO_3_ (0.63 mmol), and 7 mg of Pd(PPh_3_)_4_ (0.006 mmol) were suspended in a mixed solvent of toluene/H_2_O/EtOH (10 mL/5 mL/5 mL) under argon. The mixture was heated at 80 °C under Ar for 4 h. After cooling down to room temperature, the reaction was quenched with 0.6 mL of a 2M HCl aqueous solution, extracted with toluene, and washed with water and brine. The organic layer was dried over Na_2_SO_4_, filtered, and concentrated under reduced pressure. The obtained crude product was purified by column chromatography on silica gel (CH_2_Cl_2_/hexane = 1:3 to 1:2, *v*/*v*) to afford **A_66_** as a green powder (30 mg, 50%). ^1^H NMR (300 MHz, CDCl_3_) *δ* 8.76 (s, 2H), 8.55 (d, *J* = 10.1 Hz, 4H), 8.04 (dd, *J* = 3.7 Hz, 2H), 7.79 (d, *J* = 10.0 Hz, 4H), 7.53 (d, *J* = 3.8 Hz, 4H), 1.59 (s, 18H). ^13^C NMR (101 MHz, CDCl_3_) δ 176.71, 152.62, 151.30, 150.71, 145.95, 140.01, 138.25, 136.21, 135.29, 126.38, 118.53, 112.35, 41.00, 30.35. HR–MS (ESI, positive): *m*/*z* calcd for [M + H]^+^ 571.2856, found 571.2854.

Synthesis of **azulene-tTAP-Br**. Quantities of 65 mg of 2Br-tTAP (0.137 mmol), 36 mg of 2-(4,4,5,5-teramethyl-1,3,2-dioxaborolan-2-yl)azulene (0.137 mmol), 267 mg of Cs_2_CO_3_ (0.819 mmol), and 9 mg of Pd(PPh_3_)_4_ (0.008 mmol) were added to a 100 mL two-necked flask and flushed with argon. A mixed solvent of toluene/EtOH/H_2_O (10 mL/5 mL/5 mL) was added into the flask, and the mixture was heated at 80 °C under Ar for 7 h. The reaction was quenched with 0.6 mL of a 2M HCl aqueous solution, extracted with toluene, and washed with water and brine. The organic layer was dried over Na_2_SO_4_, filtered, and concentrated under reduced pressure. The obtained crude product was purified by column chromatography on silica gel (CH_2_Cl_2_/hexane = 1:4 to 1:2, *v*/*v*) to afford **azulene-tTAP-Br** as a brown powder (35 mg, 45%). ^1^H NMR (300 MHz, CD_2_Cl_2_) *δ* 9.08 (s, 1H), 8.58–8.54 (m, 3H), 8.50 (d, *J* = 9.6 Hz, 2H), 7.64 (dd, *J* = 9.9 Hz, 1H), 7.25 (dd, *J* = 9.8 Hz, 2H), 1.75 (s, 9H), 1.69 (s, 9H). ^13^C NMR (101 MHz, CD_2_Cl_2_) δ 176.85, 153.72, 151.27, 146.38, 144.86, 142.60, 140.90, 138.69, 138.40, 134.46, 124.23, 120.19, 112.26, 41.35, 41.06, 30.58, 30.45. HR–MS (ESI, positive): *m*/*z* calcd for [M + H]^+^ 523.1492, found 523.1491.

Synthesis of **A_26_**. Quantities of 35 mg of **azulene-tTAP-Br** (0.067 mmol), 17 mg of 6-(4,4,5,5-teramethyl-1,3,2-dioxaborolan-2-yl) azulene (0.067 mmol), 130 mg of Cs_2_CO_3_ (0.41 mmol), and 4 mg of Pd(PPh_3_)_4_ (0.004 mmol) were added to a 50 mL two-necked flask and flushed with argon. A mixed solvent of toluene/H_2_O/EtOH (10 mL/5 mL/5 mL) was added into the flask, and the mixture was heated at 80 °C under Ar for 4 h. After cooling down to room temperature, the reaction was quenched with 0.6 mL of a 2 M HCl aqueous solution, extracted with toluene, and washed with water and brine. The organic layer was dried over Na_2_SO_4_, filtered, and concentrated under reduced pressure. The obtained crude product was purified by column chromatography on silica gel (CH_2_Cl_2_/hexane = 1:2 to 1:1, *v*/*v*) to afford **A_26_** as a green powder (19 mg, 48%). ^1^H NMR (300 MHz, CD_2_Cl_2_) *δ* 9.14 (s, 1H), 8.73 (s, 1H), 8.59 (s, 2H), 8.51–8.58 (m, 4H), 8.02 (dd, *J* = 4.0 Hz, 1H), 7.82 (d, *J* = 10.3 Hz, 2H), 7.66 (dd, *J* = 10.1 Hz, 1H), 7.52 (d, *J* = 3.8 Hz, 2H), 7.27 (dd, *J* = 9.7 Hz, 2H), 1.77 (s, 9H), 1.60 (s, 9H). HR–MS (ESI, positive): *m*/*z* calcd for [M + H]^+^ 571.2856, found 571.2855.

## 4. Conclusions

In this work, we successfully synthesized and characterized a series of azulene–TAP triads to systematically study how connectivity affects their electronic properties. UV-Vis absorption and electrochemical analyses revealed that the connectivity pattern between the central TAP and azulene rings plays a decisive role in governing effective π-conjugation, molecular planarity, and optical and redox behavior. Specifically, triads **A_22_** and **A_26_**, in which the TAP core is directly connected through the electron-rich five-membered ring, exhibit stronger conjugation and distinct protonation-induced color changes, in contrast to **A_66_**, which is attached via the electron-deficient seven-membered ring. This pronounced acid-responsive behavior arises from the formation of aromatic 6π-electron tropylium cations, which are effectively delocalized with the TAP core. These findings underscore the promising strategy of connectivity engineering in azulene-based π-systems and demonstrate their strong potential as tunable building blocks for the development of advanced optoelectronic and stimuli-responsive materials.

## Data Availability

The original contributions presented in this study are included in the article and Supplementary Material. Further inquiries can be directed to the corresponding authors.

## Supplementary Materials

The following supporting information can be downloaded at https://www.mdpi.com/article/10.3390/molecules31010002/s1, Chart S1. Chemical structure of azulene. Figures S1 and S2: The gradual spectral and solution color evolution of **A_26_** and **A_66_** at a concentration of 10^−5^ M in CH_2_Cl_2_ with the successive addition of TFA (*v*/*v*) and after neutralization with TEA. Figure S3: Cyclic voltammograms and differential pulse voltammograms of **A_22_**, **A_26_**, **A_66_**, and **azulene**. Figure S4: Computed absorption spectra and oscillator strengths of **A_22_**, **A_26_**, and **A_66_**. Figure S5: The molecular orbitals involved in the main transitions of (a) **A_22_**, (b) **A_26_**, and (c) **A_66_**. Figure S6: Computed absorption spectra of **A_22_**, **A_26_**, and **A_66_** in vacuum, water, DSMO, and methanol. Figure S7: Computed absorption spectra and oscillator strengths of the protonated forms of **A_22_**, **A_26_**, and **A_66_**. Figure S8: The molecular orbitals involved in the main transitions of (a) protonated **A_22_**, (b) protonated **A_26_**, and (c) protonated **A_66_**. Figures S9–S14: ^1^H NMR spectra of **A_22_**, **A_26_**, **A_66_**, and **azulene-tTAP-Br**, and ^13^C NMR spectra of **A_66_** and **azulene-tTAP-Br**; Figures S15–S18: MS spectra of **A_22_**, **A_26_**, **A_66_**, and **azulene-tTAP-Br**; Table S1: Optical data of the target compounds **A_22_**, **A_26_**, and **A_66_** together with reference compounds. Table S2: Electrochemical data of the target compounds **A_22_**, **A_26_**, and **A_66_** together with reference compounds. Tables S3–S8: Energy, wavelength, oscillator strength, and major molecular orbital contributions to transitions of triads and their protonated species that have a sizable oscillator strength.
