# Supplementary material for: Connectivity Effect on Electronic Properties of Azulene–Tetraazapyrene Triads
Source: Molecules. 2025 Dec 19;31(1):2. doi: 10.3390/molecules31010002 (PMC12786884; doi:10.3390/molecules31010002)
Supplement: Supplementary file 1 [file molecules-31-00002-s001.zip › molecules-3984619-supplementary.pdf]

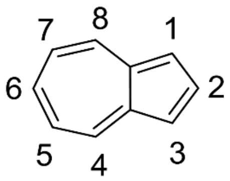

Chart S1. Chemical structure of azulene.

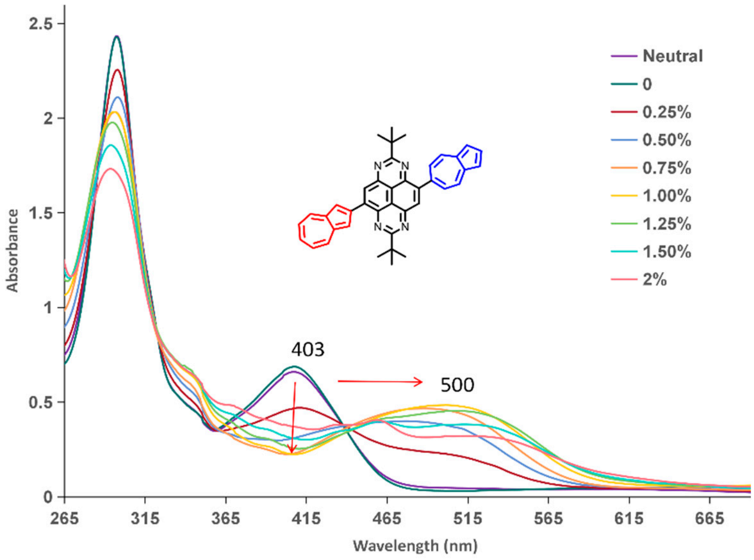

(**a**)

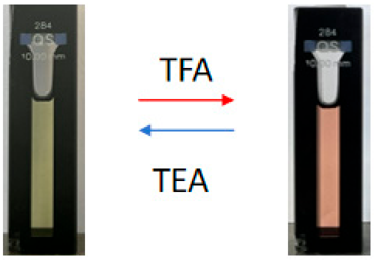

(**b**)

Figure S1. The spectral variation (**a**) and solution color evolution (**b**) of $\mathbf{A_{26}}$ at the concentration of 10^{-5} M in CH_2Cl_2 in the successive addition of TFA (v/v) and after neutralization with TEA.

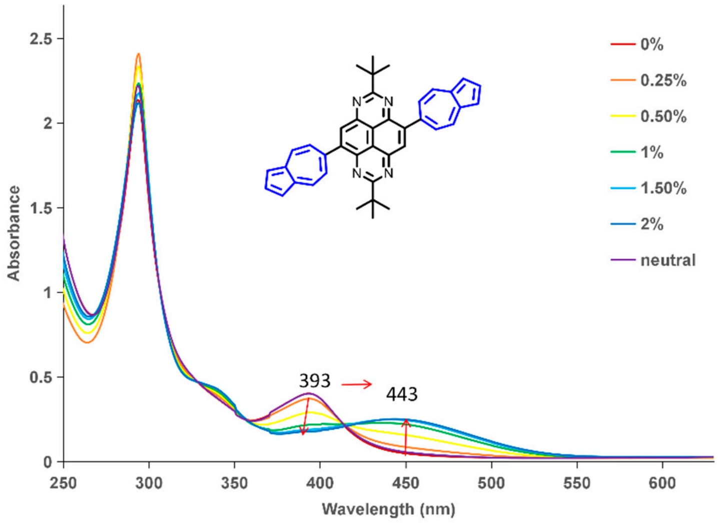

(**a**)

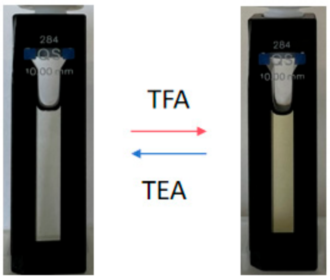

(**b**)

Figure S2. (**a**) The gradual spectral (**b**) solution color evolution of $\mathbf{A_{66}}$ at the concentration of 10^{-5} M in CH_2Cl_2 in the successive addition of TFA (v/v) and after neutralization with TEA.

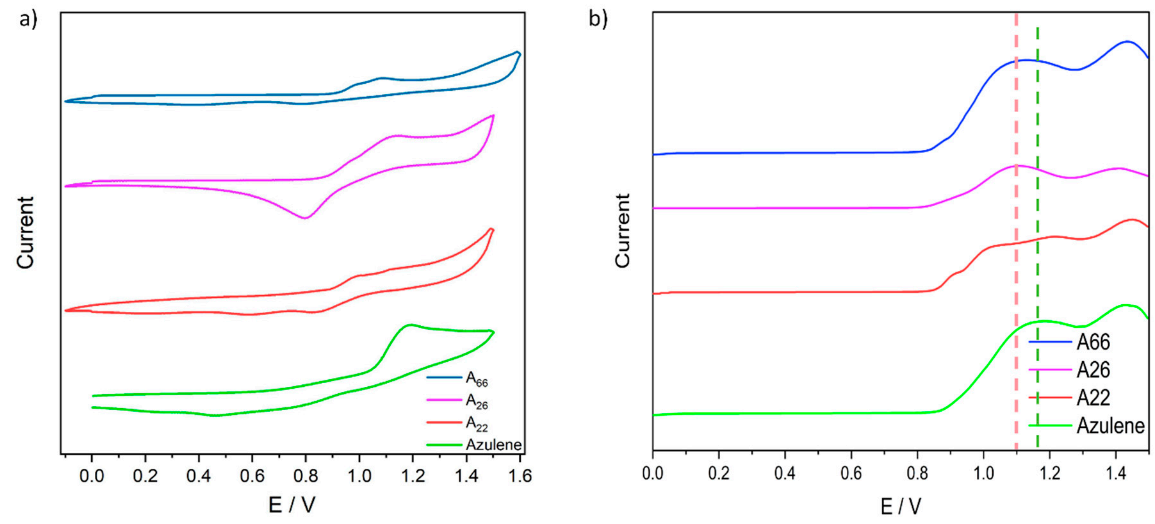

Figure S3. a) Cyclic voltammograms and b) Differential pulse voltammograms of **A_{22}** (red), **A_{26}** (purple), **A_{66}** (blue), **Azulene** (green) were measured in dichloromethane solution containing 0.1 M $TBAPF_6$ as the supporting electrolyte at r.t., Pt working electrode and Ag/AgCl reference electrode at the scan rate of 100 mV s^{-1}.

Table S1. Optical data of the target compounds **A_{22}**, **A_{26}** and **A_{66}** together with reference compounds. (c = 1×10^{-5} M) in CH_2Cl_2 at r.t.

Compounds	λ_{max}^{abs} /nm (ε / 10^5 cm^{-1} M^{-1})
**azulene**	271 (0.35), 280 (0.34), 323 (0.029), 337 (0.041)
**tTAP**	298 (0.092), 310 (0.1), 358 (0.037), 378 (0.05)
**A_{22}**	303 (0.62), 409 (0.24), 430 (0.21)
**A_{26}**	296 (0.61), 403 (0.16)
**A_{66}**	292 (0.59), 391 (0.11)

Table S2. Electrochemical data of the target compounds **A_{22}**, **A_{26}** and **A_{66}** together with reference compounds. Redox potentials (V) vs. Ag / AgCl in CH_2Cl_2.

Compounds	E_{pa} (V)	E_{ox} (V)	$E_{1/2}^{red1}$ (V)	$E_{1/2}^{red2}$ (V)	E_{red} (V)	HOMO (eV) [a]	LUMO (eV) [a]
azulene	1.18	1.05				-5.32	
**tTAP**			-1.12		-1.05		-3.22
**A_{22}**	0.98	0.89	-0.9	-1.30[b]	-0.80	-5.16	-3.47
**A_{26}**	1.09	0.87	-0.95	-1.31	-0.83	-5.14	-3.44
**A_{66}**	1.10	0.88	-1.0	-1.36	-0.89	-5.15	-3.38

[a] $E_{HOMO} = -e\,(E_{ox} - 0.53 + 4.8)$, $E_{LUMO} = -e\,(E_{red} - 0.53 + 4.8)$, E_{ox} = the onset oxidation potential, E_{red} = the onset reduction potential, the redox potential of Fc/Fc^+ is measured at 0.53 V relative to Ag/AgCl in CH_2Cl_2. [b] The cathodic peak potential.

TD-DFT calculations

Table S3. Energy, wavelength, oscillator strength, and major molecular orbital contributions to transitions of $\mathbf{A_{22}}$ that have a sizable oscillator strength.

State	Wavelength (nm)	Oscillator strength (-)	Contributions
S6	454.3	0.3700	H-2→L (72%), H-4→L (24%)
S7	435.7	0.2050	H-4→L (71%), H-2→L (24%)
S12	367.6	0.3742	H-3→L+1 (72%)
S13	355.7	0.0336	H-5→L (77%)
S15	336.6	0.0450	H-2→L+2 (52%), H→L+3 (23%), H-3→L+1 (12%)
S19	308.2	0.0610	H-4→L+2 (63%), H-6→L+1 (12%)
S20	307.8	0.4160	H-7→L (57%), H-1→L+4 (17%)
S23	305.0	0.0557	H-9→L (78%)
S26	294.7	0.5853	H-2→L+3 (50%), H-6→L+1 (14%)
S27	292.0	0.1014	H-6→L+1 (62%)
S29	285.4	0.1588	H-1→L+6 (58%), H-3→L+4 (11%)
S32	280.9	0.1295	H-1→L+6 (35%), H-2→L+5 (24%), H-3→L+4 (21%)
S33	279.4	0.5335	H-4→L+3 (39%), H-4→L+2 (11%)
S36	277.6	0.3890	H-5→L+2 (42%), H-4→L+3 (21%)
S37	271.2	0.2691	H-5→L+2 (41%), H-2→L+3 (15%)
S40	270.2	0.0271	H-1→L+7 (91%)

Table S4. Energy, wavelength, oscillator strength, and major molecular orbital contributions to transitions of **A_{26}** that have a sizable oscillator strength.

State	Wavelength (nm)	Oscillator strength (-)	Contributions
S5	453.4	0.2699	H-2→L (81%), H-4→L (15%)
S7	428.3	0.1730	H-4→L (46%), H-3→L (41%)
S11	363.5	0.1457	H-2→L+1 (70%), H-1→L+4 (19%)
S12	354.1	0.0634	H-5→L (56%), H→L+3 (23%)
S13	351.9	0.0152	H→L+3 (40%), H-5→L (29%), H-3→L+1 (12%)
S14	347.9	0.0394	H-3→L+1 (34%), H→L+3 (34%), H→L+5 (24%)
S15	340.2	0.0109	H-1→L+3 (92%)
S20	316.0	0.2994	H-7→L (58%)
S21	304.2	0.0229	H-9→L (76%)
S22	303.8	0.0506	H-4→L+2 (57%), H-4→L+3 (13%), H-9→L (11%)
S23	300.4	0.1949	H-8→L (65%)
S25	295.9	0.1648	H-5→L+1 (48%), H-2→L+3 (32%)
S27	292.6	0.1967	H-5→L+1 (42%), H-2→L+3 (30%)
S28	288.4	0.0535	H-1→L+6 (76%)
S30	283.9	0.4428	H-3→L+3 (35%), H-7→L+1 (15%), H→L+5 (13%)
S31	281.4	0.0789	H-2→L+4 (60%)

Table S5. Energy, wavelength, oscillator strength, and major molecular orbital contributions to transitions of **A_{66}** that have a sizable oscillator strength.

State	Wavelength (nm)	Oscillator strength (-)	Contributions
S6	436.6	0.2879	H-2→L (82%), H-3→L (13%)
S13	350.2	0.1367	H-5→L (87%)
S14	347.3	0.0148	H-2→L+1 (49%), H→L+5 (29%)
S15	346.2	0.0404	H-3→L+1 (47%), H-1→L+4 (30%)
S16	323.1	0.0461	H-2→L+2 (42%), H-6→L (22%), H-7→L (16%)
S17	319.1	0.0264	H-3→L+2 (46%), H-2→L+1 (26%)
S18	314.4	0.2556	H-3→L+1 (20%), H-6→L (19%), H-4→L+1 (14%), H-3→L+2 (11%)
S19	312.3	0.0276	H-8→L (86%)
S21	308.7	0.0427	H-4→L+1 (77%)
S27	288.7	0.4760	H-2→L+3 (56%)
S28	287.0	0.2540	H-3→L+3 (36%), H-5→L+1 (23%)
S34	275.8	0.8778	H-2→L+3 (21%), H-6→L+2 (18%), H-7→L+2 (14%), H-8→L+1 (11%)
S36	272.9	0.0155	H-3→L+4 (17%), H-2→L+5 (16%), H-6→L+2 (15%), H-7→L+2 (12%)
S38	272.0	0.0283	H-3→L+4 (24%), H-11→L (20%), H-2→L+4 (15%), H-2→L+5 (12%)
S40	268.4	0.1138	H-5→L+2 (89%)

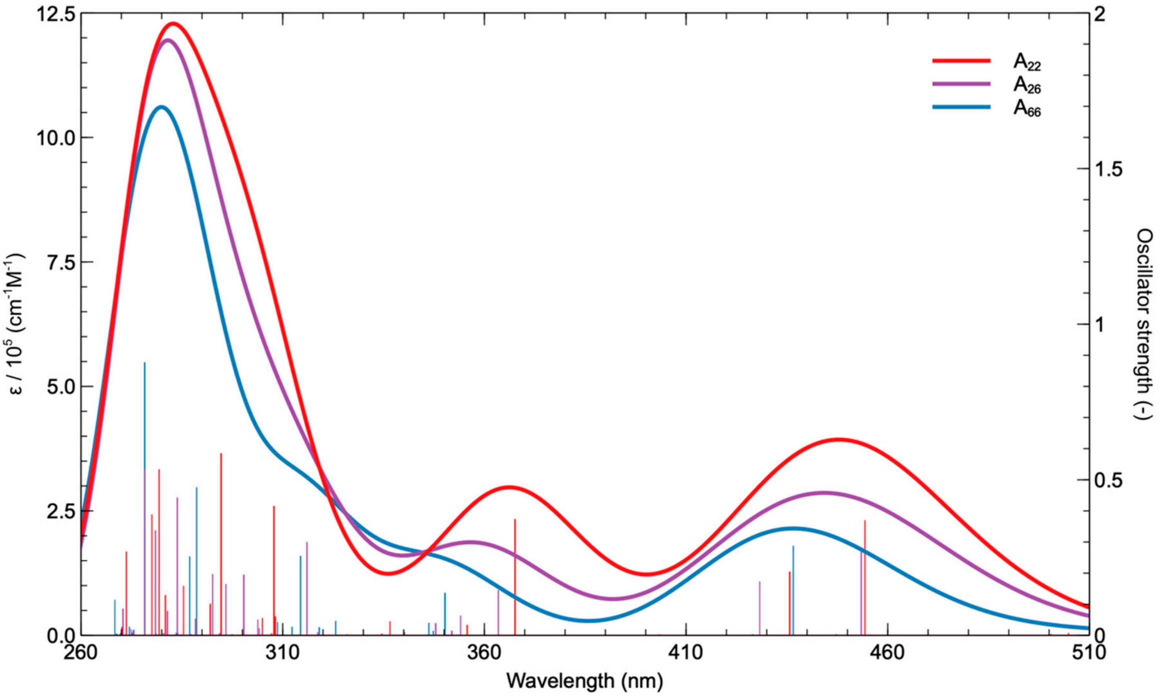

Figure S4. Computed absorption spectra and oscillator strengths of $\mathbf{A_{22}}$, $\mathbf{A_{26}}$ and $\mathbf{A_{66}}$.

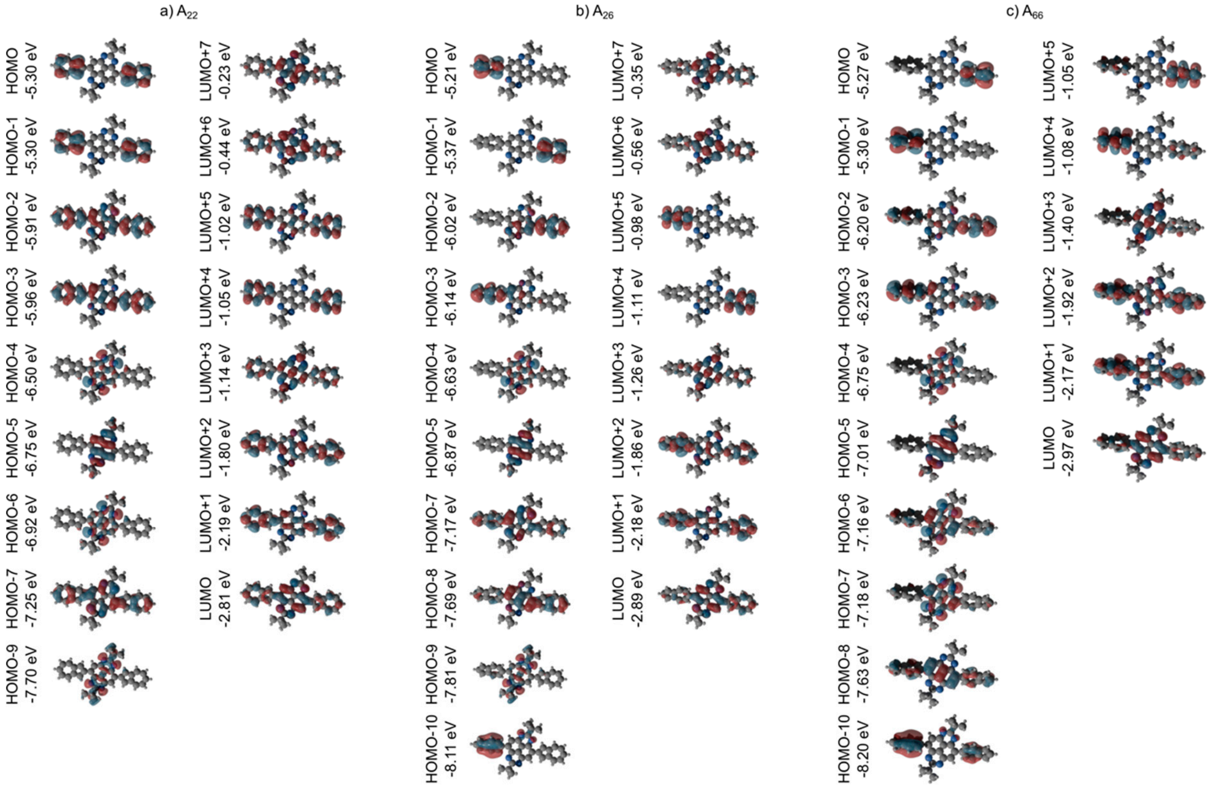

Figure S5. The molecular orbitals involved in the main transitions of a) $\mathbf{A_{22}}$, b) $\mathbf{A_{26}}$ and c) $\mathbf{A_{66}}$.

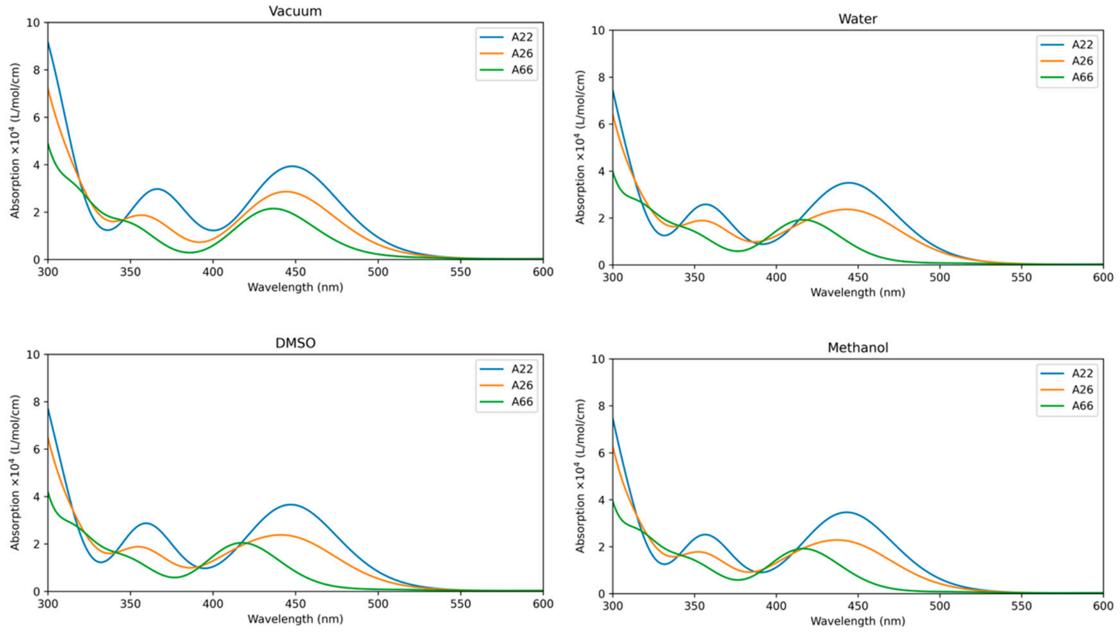

Figure S6. Computed absorption spectra of $\mathbf{A}_{22}$, $\mathbf{A}_{26}$ and $\mathbf{A}_{66}$ in vacuum, water, DSMO and methanol.

Table S6. Energy, wavelength, oscillator strength, and major molecular orbital contributions to transitions of protonated $\mathbf{A}_{22}$ that have a sizable oscillator strength.

State	Wavelength (nm)	Oscillator strength (-)	Contributions
S3	479.7	0.5064	H-1→L (92%)
S6	435.1	0.1224	H-2→L (84%)
S10	413.7	0.2230	H-1→L+3 (39%), H-3→L+1 (37%), H-3→L+2 (16%)
S13	400.1	0.8816	H-3→L+1 (51%), H-1→L+3 (36%)
S15	382.8	0.1083	H-2→L+3 (61%), H-3→L+2 (18%), H-1→L+3 (17%)
S23	340.2	0.0318	H-1→L+4 (82%), H-8→L (10%)
S28	324.3	0.0680	H-2→L+4 (79%), H-8→L (13%)
S31	308.4	0.0335	H-8→L (59%), H-7→L+1 (19%)
S33	303.4	0.0231	H-7→L+1 (80%), H-8→L (12%)

Table S7. Energy, wavelength, oscillator strength, and major molecular orbital contributions to transitions of protonated $\mathbf{A}_{26}$ that have a sizable oscillator strength.

State	Wavelength (nm)	Oscillator strength (-)	Contributions
S3	461.2	0.3471	H-1→L (87%)
S6	429.9	0.1320	H→L+2 (53%), H-2→L (22%), H-1→L+1 (16%)
S7	419.0	0.2165	H-2→L (51%), H→L+2 (11%)
S8	412.2	0.0198	H-2→L+1 (72%)
S12	402.2	0.1886	H-1→L+3 (48%), H-2→L (11%)
S14	385.8	0.0338	H-4→L (46%), H-2→L+2 (29%)
S16	379.4	0.0559	H-2→L+3 (45%), H→L+4 (22%)
S17	374.7	0.1248	H-3→L+2 (40%), H-4→L+1 (23%), H→L+4 (22%)
S18	366.0	0.3410	H-4→L+1 (56%), H-3→L+2 (33%)
S22	342.0	0.0193	H-1→L+4 (55%), H-6→L (18%), H-5→L (11%)
S25	334.6	0.0103	H-4→L+3 (41%), H-2→L+4 (16%), H-1→L+4 (14%)
S27	325.8	0.0147	H-7→L (32%), H-8→L (17%), H-2→L+4 (16%)
S28	324.3	0.0239	H-7→L (37%), H-2→L+4 (27%), H-6→L (10%)
S29	323.8	0.0419	H-6→L+1 (26%), H-6→L+2 (17%), H-5→L+2 (14%), H-2→L+4 (13%)

Table S8. Energy, wavelength, oscillator strength, and major molecular orbital contributions to transitions of protonated **A$_{66}$** that have a sizable oscillator strength.

State	Wavelength (nm)	Oscillator strength (-)	Contributions
S3	439.8	0.1501	H-1→L (91%)
S5	429.9	0.0813	H→L+2 (79%)
S11	392.3	0.2187	H-3→L (52%), H-2→L (22%)
S12	388.4	0.1194	H-3→L+1 (50%), H-2→L+1 (20%), H-3→L (11%)
S13	382.3	0.0163	H→L+4 (51%), H-2→L+1 (26%), H-3→L+1 (12%)
S17	369.2	0.0124	H-2→L+3 (66%), H-3→L+3 (16%)
S19	351.7	0.4396	H-4→L+1 (71%)
S23	339.3	0.1021	H-1→L+4 (44%), H-4→L+2 (24%), H-4→L+3 (17%)
S24	336.7	0.0385	H-4→L+2 (37%), H-4→L+3 (26%)
S25	332.1	0.0104	H-5→L (39%), H-4→L+3 (27%)
S29	316.2	0.0157	H-5→L+1 (28%), H-7→L (27%), H-8→L (17%)
S30	313.1	0.1071	H-5→L+2 (27%), H-7→L (24%), H-5→L+3 (10%)
S31	312.8	0.1541	H-6→L+2 (54%), H-5→L+2 (15%)
S32	312.1	0.1434	H-5→L+3 (24%), H-5→L+1 (15%), H-5→L+2 (14%)
S33	309.3	0.0121	H-6→L+3 (62%)
S34	307.8	0.0283	H-7→L+1 (31%), H-6→L+3 (22%), H-3→L+4 (16%)
S36	303.1	0.0122	H-8→L+1 (69%)
S37	302.1	0.0325	H-7→L+1 (38%), H-3→L+4 (18%)
S38	299.5	0.0108	H-4→L+4 (60%)

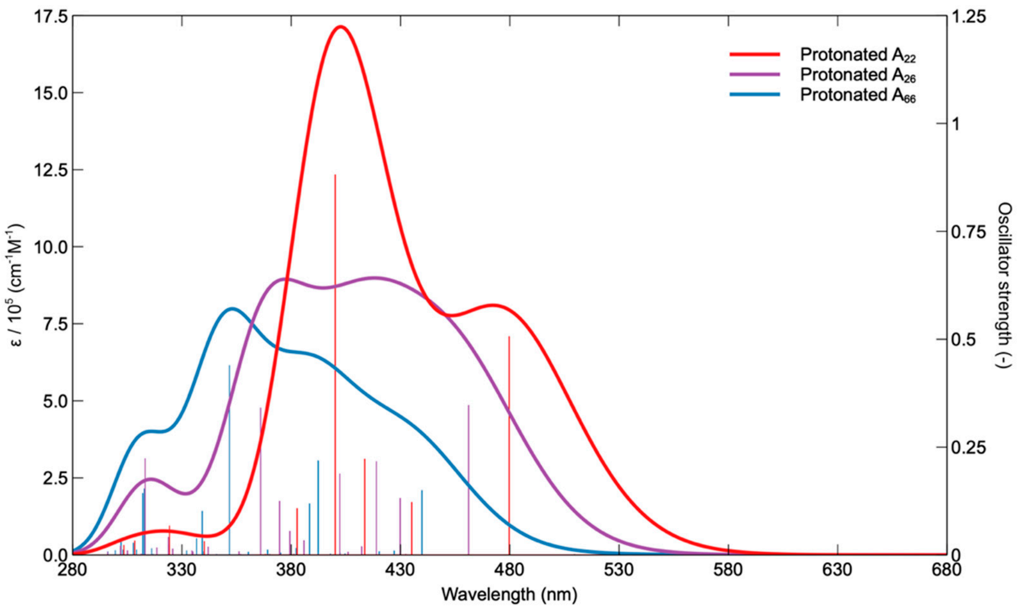

Figure S7. Computed absorption spectra and oscillator strengths of the protonated forms of **A$_{22}$**, **A$_{26}$** and **A$_{66}$**.

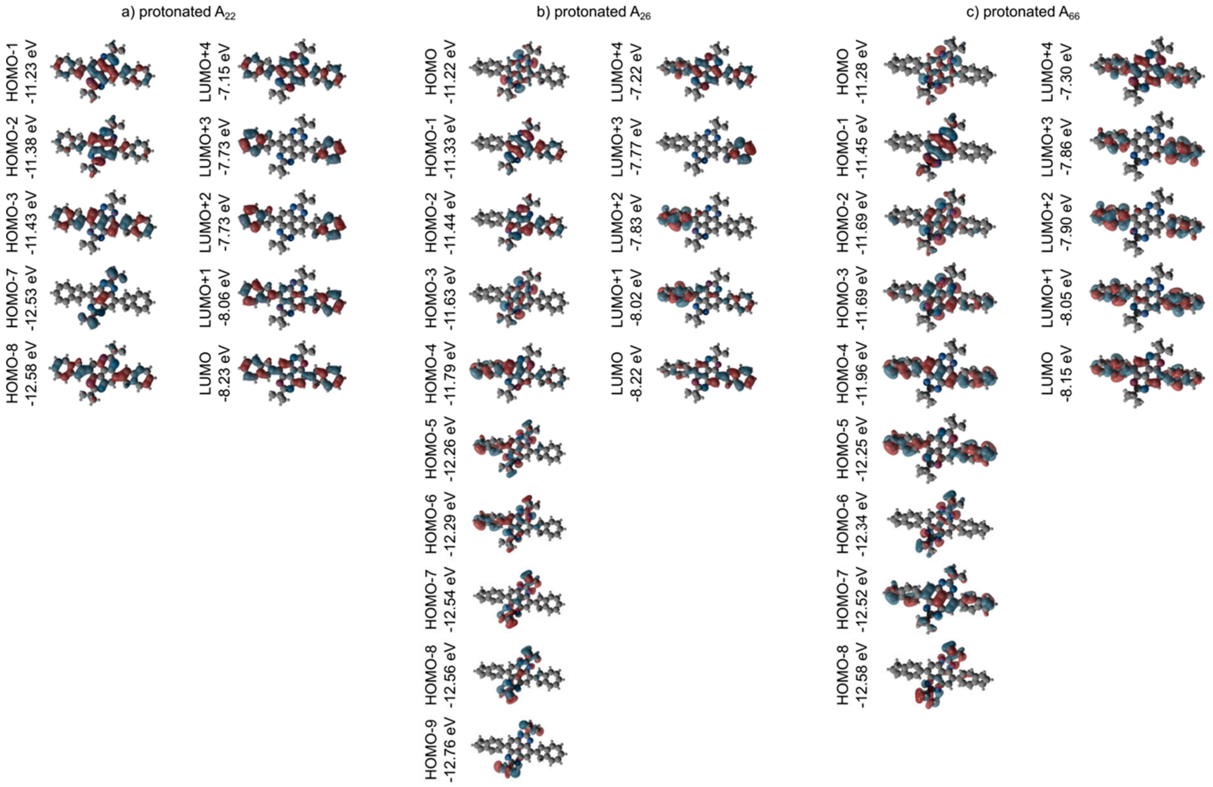

Figure S8. The molecular orbitals involved in the main transitions of a) protonated $\mathbf{A_{22}}$, b) protonated $\mathbf{A_{26}}$ and c) protonated $\mathbf{A_{66}}$.

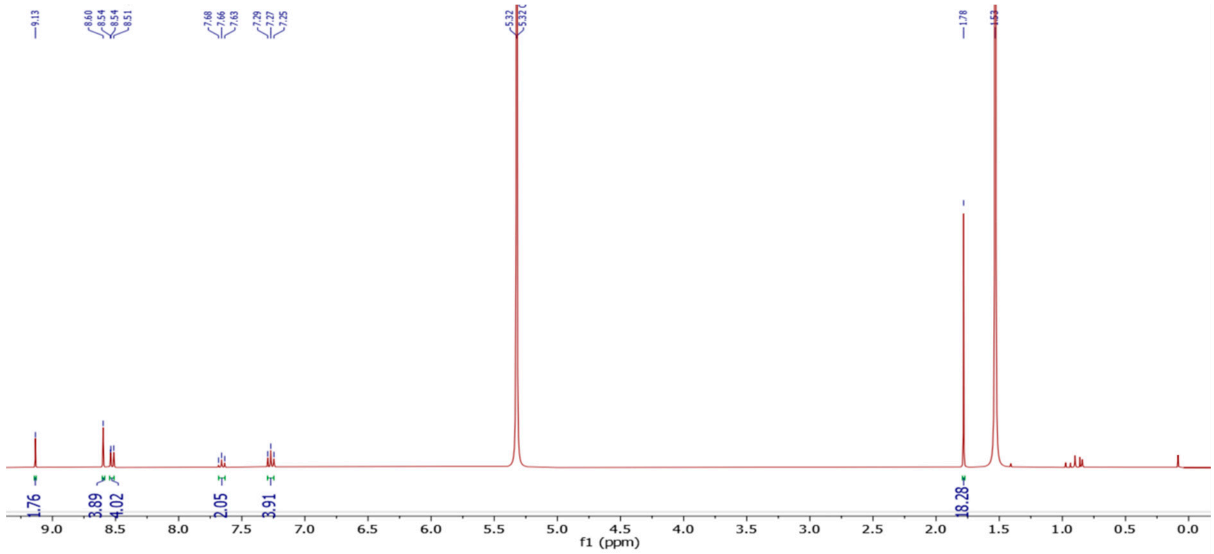

Figure S9. ^{1}H NMR spectrum of **A22** in CD_2Cl_2.

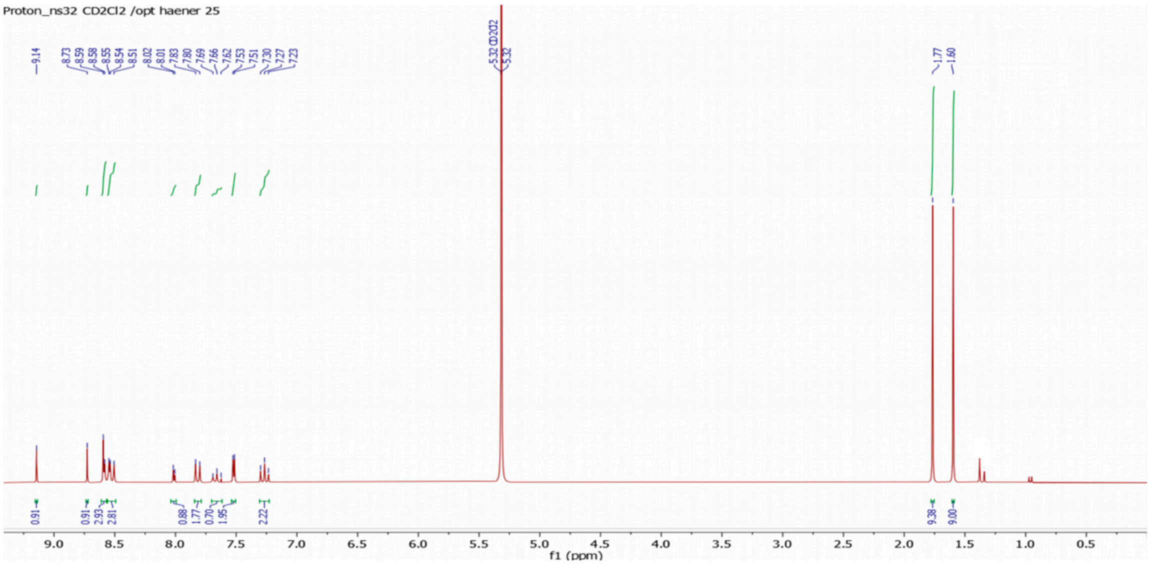

Figure S10. ^{1}H NMR spectrum of **A26** in CD_2Cl_2.

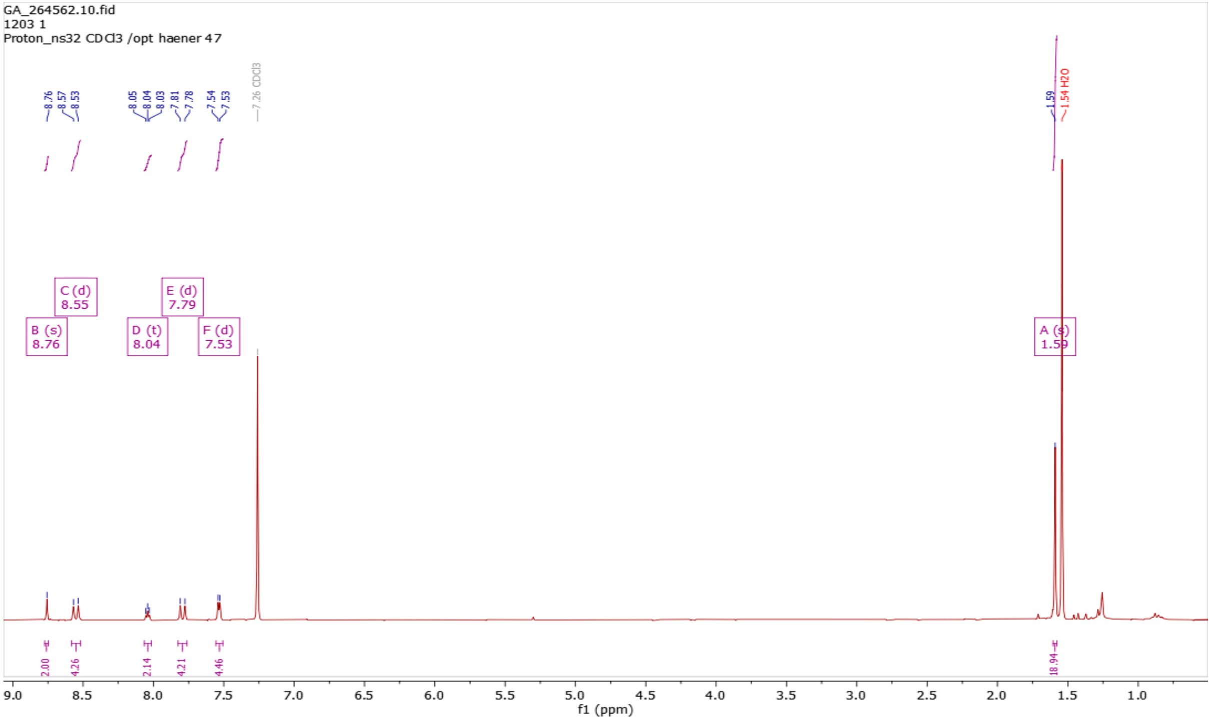

Figure S11. ^{1}H NMR spectrum of **A_{66}** in $CDCl_3$.

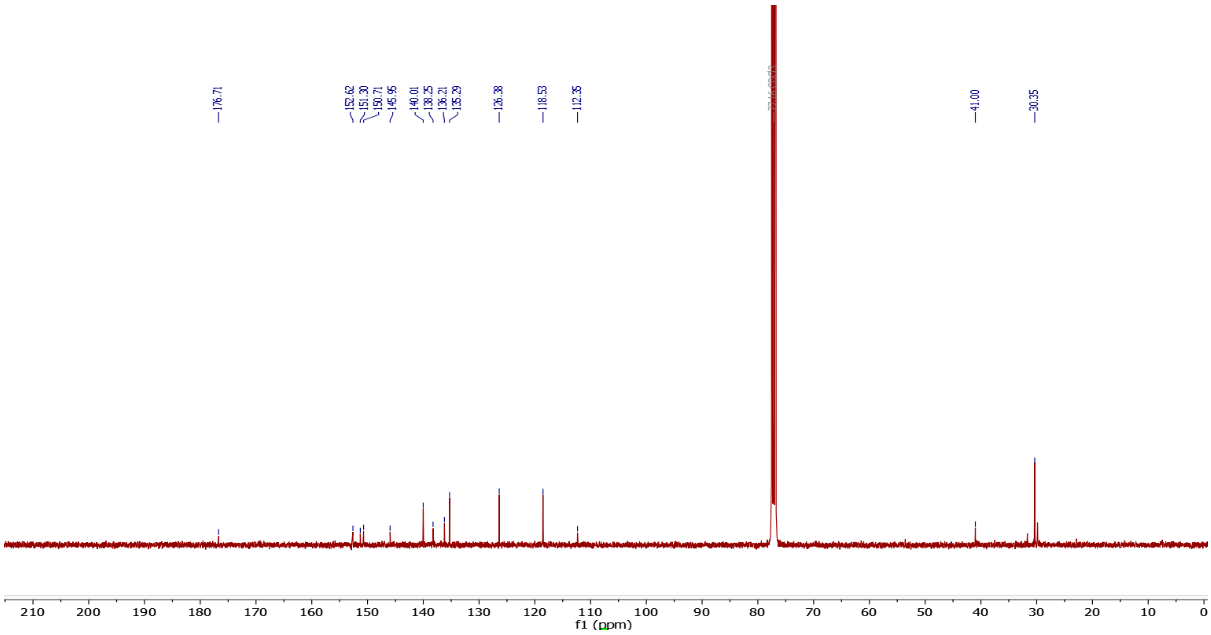

Figure S12. ^{13}C NMR spectrum of **A_{66}** in $CDCl_3$.

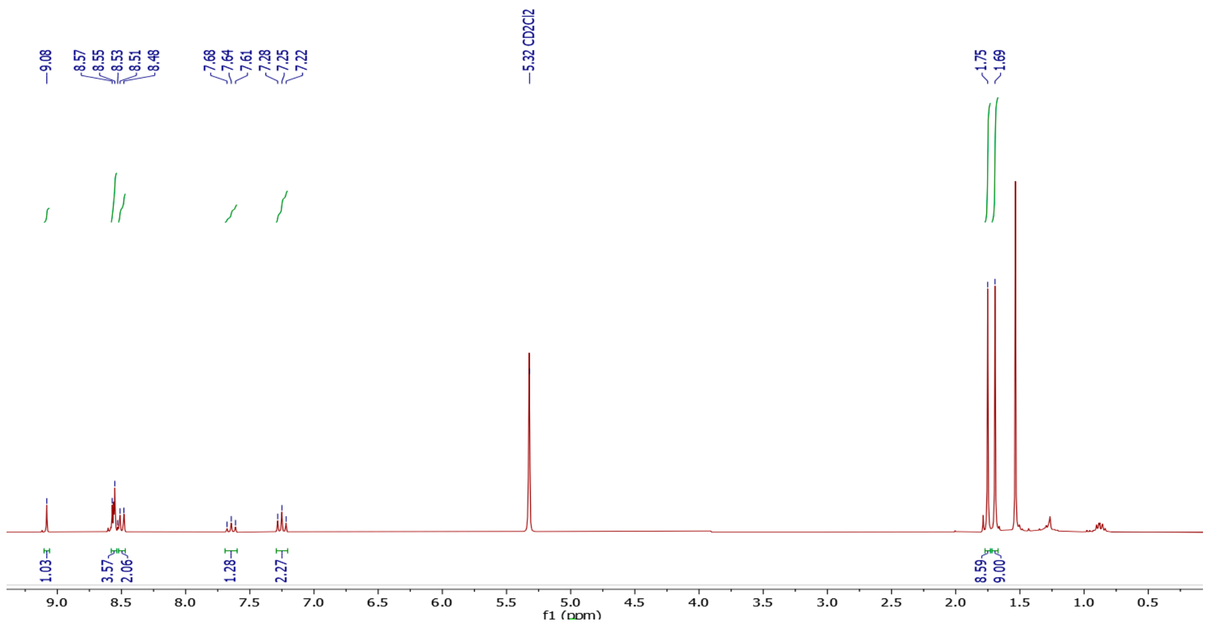

Figure S13. ^{1}H NMR spectrum of **azulene-tTAP-Br** in CD_2Cl_2.

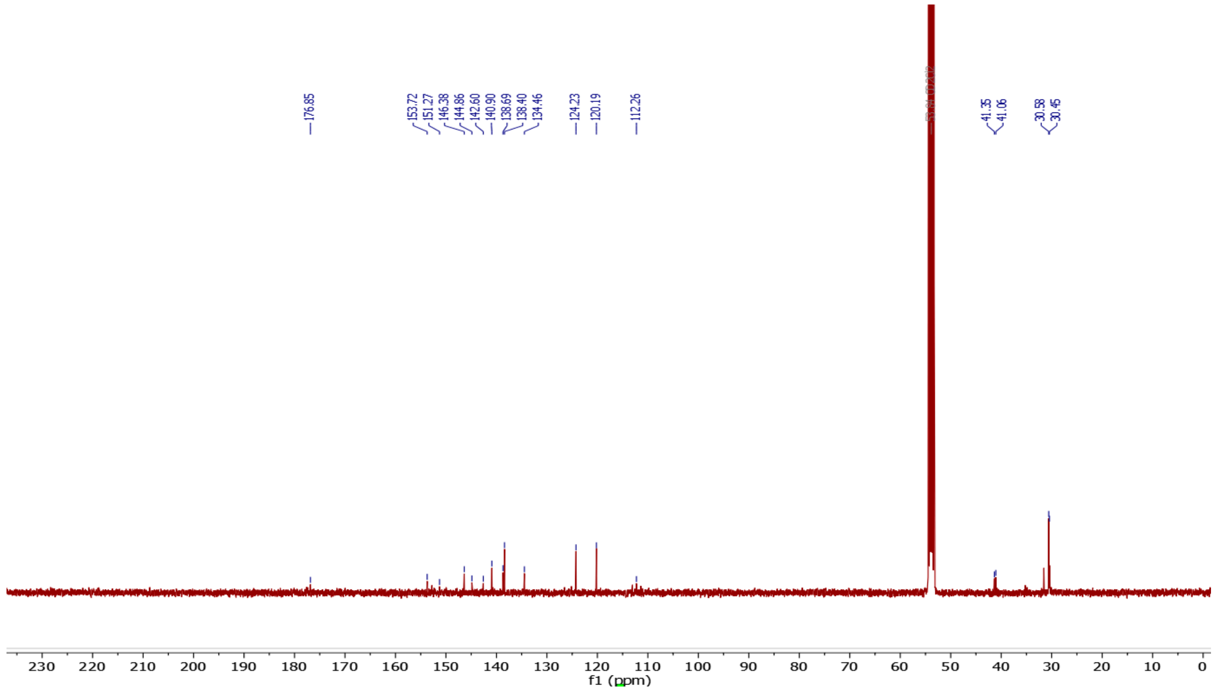

Figure S14. ^{13}C NMR spectrum of **azulene-tTAP-Br** in CD_2Cl_2.

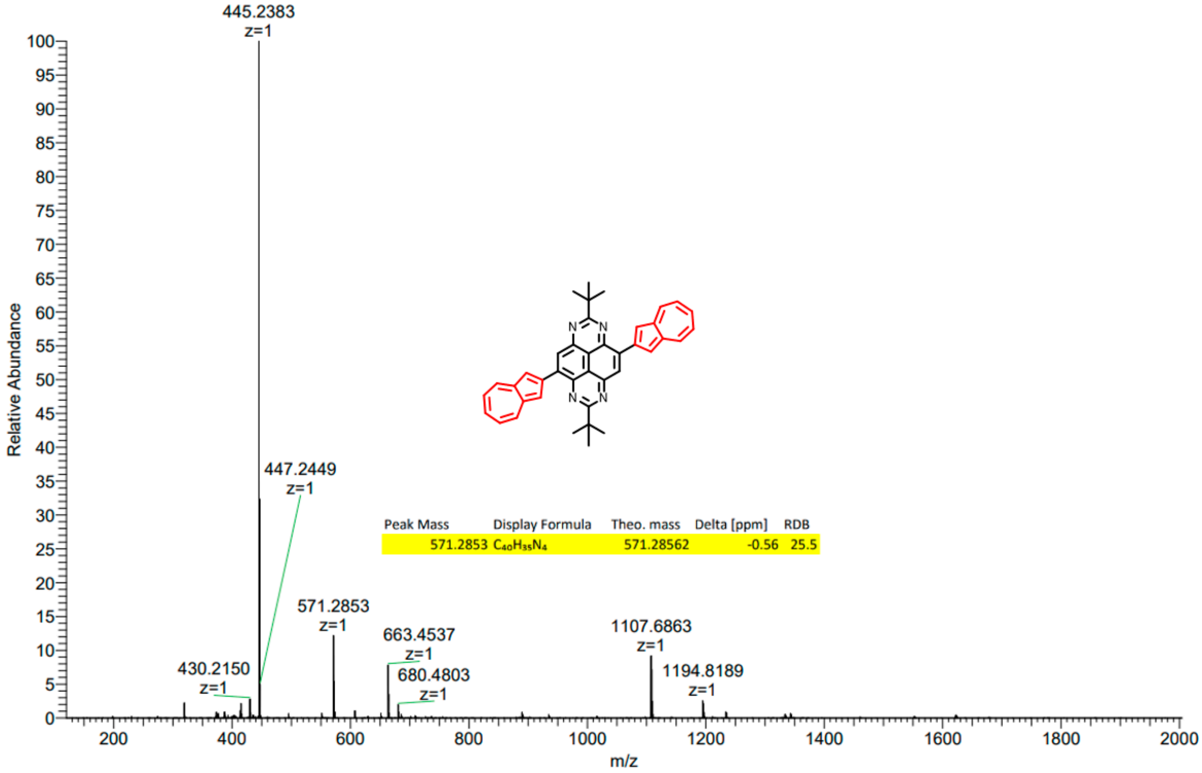

Figure S15. MS spectrum of **A**$_{22}$.

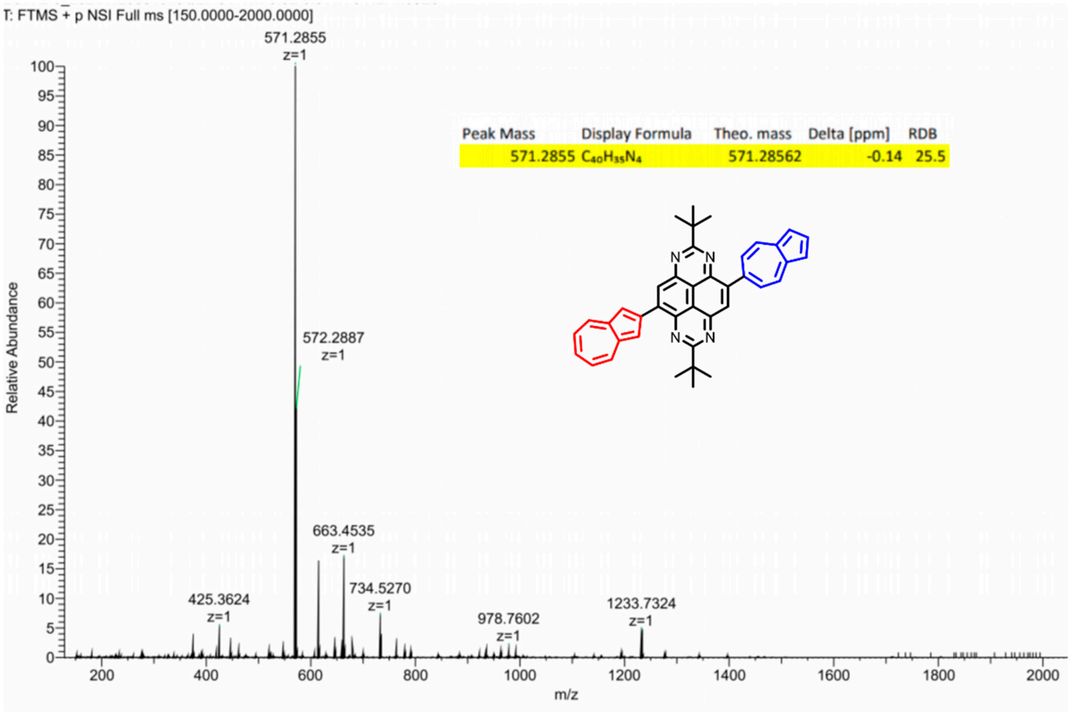

Figure S16. MS spectrum of **A**$_{26}$.

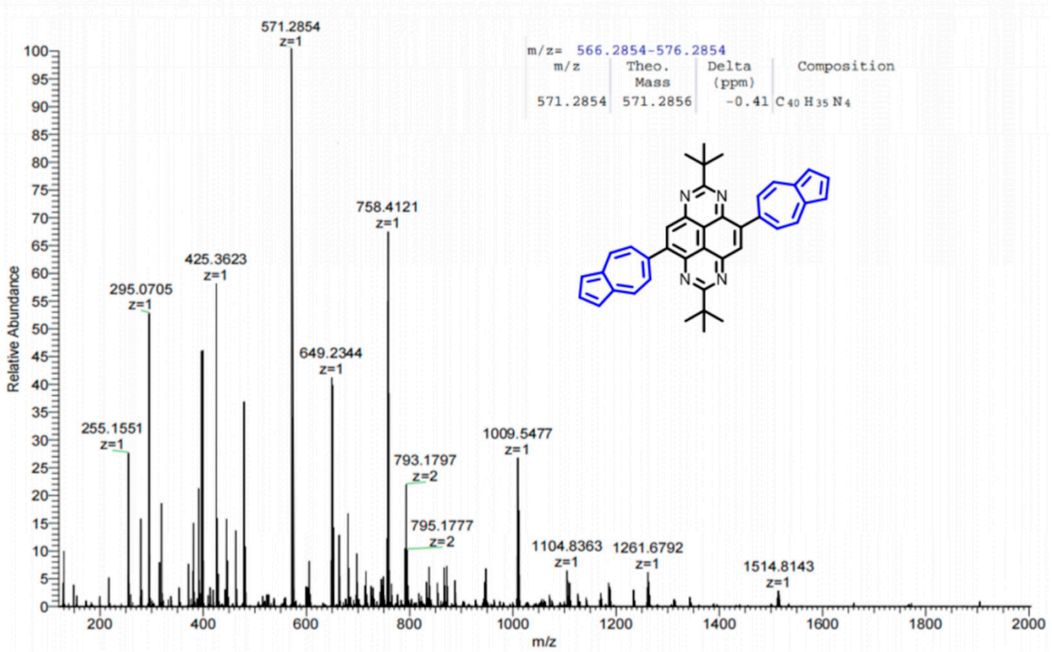

Figure S17. MS spectrum of **A_{66}**.

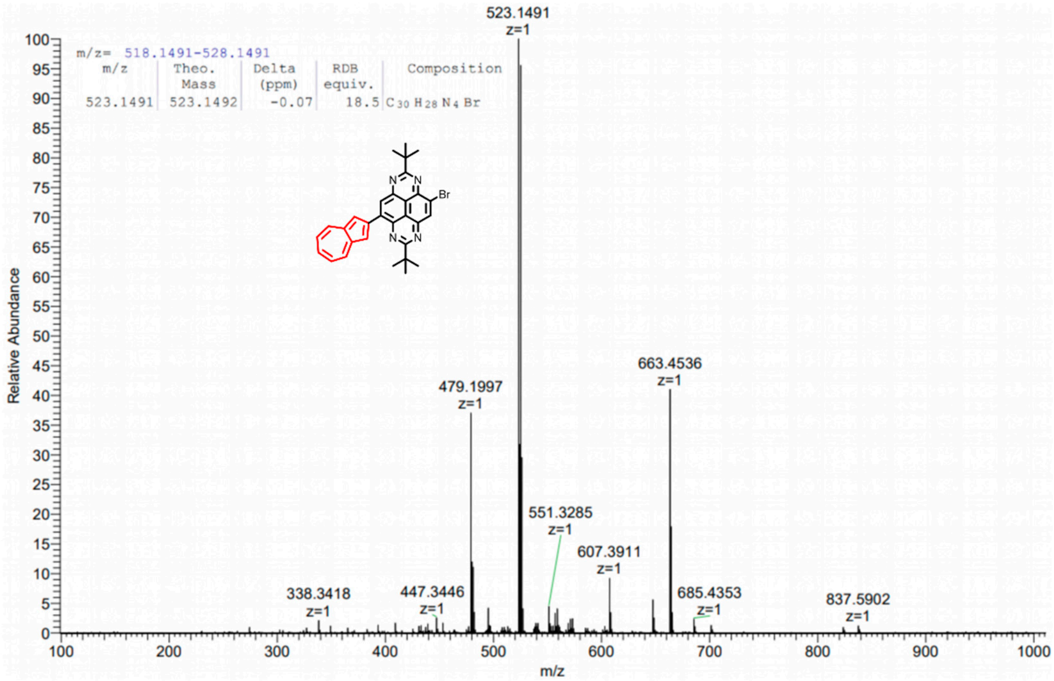

Figure S18. MS spectrum of **azulene-tTAP-Br**.
